# Predictors of Change in Osteoporosis Knowledge, Health Beliefs, and Self-Efficacy After an Education Intervention

**DOI:** 10.1093/geroni/igaa057.1466

**Published:** 2020-12-16

**Authors:** Philippe Ronel Labrias, Richard Feinn, Shanthi Johnson, Katherine McLeod

**Affiliations:** 1 Frank H. Netter MD School of Medicine, Hamden, Connecticut, United States; 2 Frank H. Netter MD School of Medicine Quinnipiac University, Hamden, Connecticut, United States; 3 University of Alberta, Edmonton, Alberta, Canada

## Abstract

Education interventions that increase osteoporosis knowledge and address health beliefs and self-efficacy help older adults make informed decisions to prevent and manage the disease. The aim of this study was to determine if clinical risk factors for osteoporosis moderate the effect of a multifaceted education intervention on osteoporosis knowledge, health beliefs, and self-efficacy. Patients 50 years and older with no prior diagnosis of or treatment for osteoporosis were referred by their primary care provider for bone mineral density testing by DXA and randomized to an osteoporosis education intervention group (n = 102) or usual care group (n = 101). Demographic and health history questionnaires, and validated tools to assess osteoporosis knowledge, health beliefs and self-efficacy were completed at baseline and 6-month follow-up. Results of the linear mixed-effects model showed a significant interaction with younger age (p=.024) on self-efficacy among patients in the intervention group compared to the usual care group. Patients with higher BMI had greater perceived health motivation (p=.026) in the intervention group. Compared to the usual care group, patients in the intervention group with higher vitamin D intake had greater perceived exercise (p=.020) and calcium benefits (p=.012) and those with a family history of osteoporosis had greater perceived susceptibility to osteoporosis (p=.045). By understanding the key factors that predict change in knowledge, health beliefs and self-efficacy after an education intervention compared to usual care, we can better tailor interventions to enhance prevention and management of osteoporosis.

